# *Mycobacterium tuberculosis* and *Pseudoramibacter alactolyticus* coinfection in brain after dental extraction: A case report

**DOI:** 10.1097/MD.0000000000018289

**Published:** 2019-12-16

**Authors:** Yixin Liao, Fan Wu, Fahui Dai, Qin Huang, Yanling Feng, Yun Ling, Hongzhou Lu

**Affiliations:** a State Key Laboratory of Chemo/Biosensing and Chemometrics, College of Biology, Hunan University, Changsha; b Eastern China Center for Pathogen Discovery and Research; c Department of Infectious Diseases; d Department of Pathology, Shanghai Public Health Clinical Center, Fudan University, Shanghai, China.

**Keywords:** 16S ribosomal RNA targeted sequencing, dental extraction, tuberculosis meningitis

## Abstract

**Introduction:**

More than 1200 different types of microbes were found in the human mouth, only some of these microorganisms were associated with intracranial bacterial infection. However, there are limited data available about the *Pseudoramibacter alactolyticus (P alactolyticus)* or *Mycobacterium tuberculosis* (*MTB*) intracranial infections oral origin.

**Patient concerns:**

Here, we reported a rarely case with *P alactolyticus* and *MTB* coinfection in central nervous after dental extraction. The 44-year-old man presented with progressive headache over the last 2 weeks and a sustained fever >39°C, with a dental extraction performed 2 days before the onset of headache.

**Diagnosis:**

*P alactolyticus* and *MTB* were confirmed by real-time polymerase chain reaction targeting the16S ribosomal RNA gene. The presence of *MTB* was also demonstrated by positive acid-fast staining of the purulent discharge.

**Interventions:**

The patient was treated by metronidazole and anti-TB treatment

**Outcomes:**

The patient fully recovered without sequela.

**Conclusion:**

In conclusion there should be awareness of the possibility of *P alactolyticus* or *MTB* intracranial infections following tooth extraction.

## 1 Introduction

The oral cavity is a major gateway to human body, and >1200 different types of microbes were found in the human mouth.^[1]^*Atopobium genomospecies C1* (53%), *Pseudoramibacter alactolyticus (P alactolyticus)* (37%), and *Streptococcus species* (33%) were the popular microbiota of the most advanced layers of dentinal caries in teeth.^[2]^ Infection is the most common complication after tooth extraction.^[3]^ Regularly prophylactic antibiotic was recommended before and after tooth extraction,^[4]^ which can decrease the risk of bacterial infection.^[5,6]^ Intracranial bacterial infection are rare but potentially deadly complications of odontogenic infections, associated with some of oral microorganisms.^[7]^ Here, we reported a rarely case with *P alactolyticus* and *Mycobacterium tuberculosis* (*MTB*) coinfection in central nervous after dental extraction.

**Figure 1:**
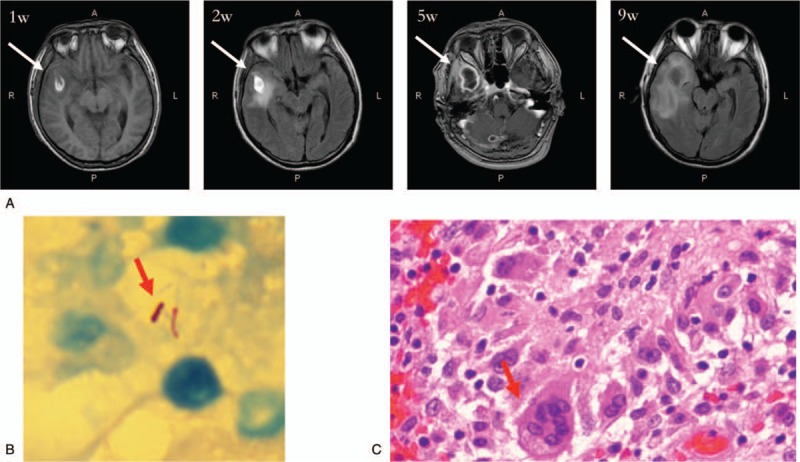
(A) Contrast-enhanced brain magnetic resonance imaging (MRI) of patient on 1, 2, 5, and 9 weeks after disease onset. (B) Acid-fast stain with bacteria indicated by the arrow in the purulent discharge from patient's brain. (C) Hematoxylin and eosin (HE) stain with macrophages indicated by arrow in histologic sections.

In March 2018, a 44-year-old man presented to the Infectious Disease department of Shanghai Public Health Clinical Center in Shanghai, China. He presented with progressive headache during the last 2 weeks and a fever >39°C. Details of the medical history revealed a dental extraction performed 2 days before the onset of headache. A brief, prophylactic course of the antibiotic metronidazole was initiated after the dental extraction. Physical examination of the patient revealed neck stiffness and a temperature of 39.5°C. Blood analyses revealed increased white cell counts of 17.09 × 10^9^ cells per liter (L) (reference range 3.50–9.50 cells/L) and an elevated proportion of neutrophils (86.7%, reference range 40.0%–75.0%). Intracranial pressure was extremely high (350 mm H_2_O, reference range 100–180 mm H_2_O) as measured via lumbar puncture. White blood cell (WBC) counts in cerebrospinal fluid (CSF) were also elevated, measured at 900 × 10^6^ cells/L (reference range 0–8 × 10^6^). Biochemical analysis of CSF revealed low glucose (1.1 mmol/L, reference range 2.2–4.4 mmol/L), and high protein levels (2483.00 mg/L, reference range 150–450 mg/L). Normal CD4+, CD8+ T cell counts without endocrine, metabolic, or autoimmune abnormalities were found in this patient. Magnetic resonance imaging (MRI) detected a massive lesion in the patient's brain surrounded by edema (Fig. 1, Panel A, T2-weighted images at 1 week).

Following a diagnosis of bacterial meningitis, the patient was treated with piperacillin sodium/tazobactam sodium and moxifloxacin upon admission. The lesion in the brain, however, was not clearly resolved despite almost normal CSF results following antibiotic treatment (Fig. 1, Panel A, 2 and 5 weeks after admission). In week 9, the patient suffered an acute, severe headache. Enhanced MRI revealed a ruptured abscess (Fig. 1, Panel A, 9 weeks after admission). The abscess was drained during emergency brain surgery, and the recovered material and surrounding tissue were sent for laboratory examination. There was an abundance of Gram-positive bacteria in the purulent discharge drained from the brain abscess, as determined by microscopy. These samples were sent for 16S rRNA targeted sequencing.^[8,9]^ As expected, *P alactolyticus* was detected, which is associated with asymptomatic periradicular lesions and acute apical periodontitis. An unexpected finding, however, was the detection of *MTB.* The presence of *MTB* was demonstrated by positive acid-fast staining (Fig. 1, Panel B, acid-fast stain with bacteria indicated by the arrow), and real-time polymerase chain reaction (PCR) targeting the16S ribosomal RNA gene of the purulent discharge, and HE stain of granulomas with macrophages in histologic sections (Fig. 1, Panel C, HE stain with macrophages), with negative T-SPOT TB test in blood, microscopy, culture, GeneXpert in cerebrospinal fluid (CSF). Once the coinfection of *P alactolyticus* and *MTB* in the brain had been confirmed, metronidazole and anti-TB treatment (isoniazid 300 mg daily for 12 months, rifampin 450 mg daily for 12months, pyrazinamide 500 mg thrice daily for 2 months, and ethambutol 750 mg daily for 2 months) were added. Upon 12 months treatment, the patient was fully recovered without sequela. No history of TB infection before the dental extraction, and no evidence of TB infection other than central nervous in this case. The patient had normal thyroid-stimulating hormone levels and tests did not reveal anti-DNA, anti-nuclear, anti-thyroglobulin antibodies or anti-HIV.

## 2 Materials and methods

### 2.1 Ethics statement

According to hospital protocol, no formal ethics approval was required for this study. The patient agreed and provided written informed consent for publication of this report and any accompanying images.

### 2.2 16S rRNA Extraction and sequencing

Total DNA and RNA of the purulent discharge drained from the brain abscess were extracted with Invitrogen PureLink Viral RNA/DNA Mini Kit (Thermo Fisher, China) as manufacturer's instructions. Bacterial 16S rDNA sequence was amplified by reverse transcriptase PCR (RT-PCR) using Takara 1-step RT-PCR Kit (Takara, China) as manufacturer's instructions with the following pairs of primers: 27F (AGA GTT TGA TCC TGG CTC AG) and 1492R (GGT TAC CTT GTT ACG ACT T). The PCR product was purified by agarose gel electrophoresis and sequenced (by Sango Biotech Inc, Shanghai, China). *MTB* was detected by real-time PCR with the following pairs of primers: 1351TB-Fvp (ATG GCG AAC TCA AGG AGC ACA), 1351TB-Rvp (GGA CAG GCC GAG TTT GGT CAT) and Probe (5’FAM- ACA CTT TGC GGG CAC CGT AAA-3’MGB).

## 3 Discussion

The caries with periapical involvement and periodontitis were the 2 most common intraoral sources,^[7]^ that the bacteria can disseminate from these locations due to periodontitis or tooth extractions.^[7]^ Many microorganisms in caries were associated with intracranial bacterial infection, such as *Streptococcus viridans*, *Actinomyces*, *Peptostreptococcus*, *Prevotella*, *Fusobacterium*, *Aggregatibacter actinomycetemcomitans* and *Eikenella corrodens,*^[7]^*without P alactolyticus or MTB. P alactolyticus* was frequently found in birds in nature and in caries in human, less disease caused.^[10]^ It was only related with primary endodontic infections, which was detected in 76% of root-canal samples from the patients’ teeth with asymptomatic periradicular lesions by nested PCR.^[2,11]^ Oral manifestation of TB represents roughly 1% of all cases of tuberculosis; however, TB has been reported associated with pulpitis till now.^[7,12]^ The patient never suffered from any inborn or acquired Immunodeficiency-associated disease. The *P alactolyticus* and *MTB* co-infection of this case may be explained that chronic inflammation may favor localization of *MTB* in the oral cavity.^[12,13]^ The detection of *MTB* may be interesting in caries from patients and controls in the TB high-burden countries.

China was listed in the TB high-burden countries, with 9% cases of the world.^[14]^ Tuberculous meningitis (TBM) represents roughly 1% of all cases of tuberculosis, but it is disproportionately important because it kills or severely disables about half of the people affected.^[15]^ Diagnosis of TBM is often delayed by the insensitive and lengthy culture technique required for disease confirmation.^[16]^ The sensitivity of microscopy to detect of acid-fast bacilli in CSF is low (10%–20%), except for the high bacteria burden.^[16]^ Culture of *MTB* from patient CSF is more sensitive than microscopy for diagnosis (around 65%).^[16]^ Molecular genetic techniques have been improved and wildly applied in diagnostic microbiology over the last few decades,^[17]^ which represent a rapid, sensitive, and specific method for TB. The GeneXpert and the second generation GeneXpert test (Ultra) found the assay to be around 60% to 90% sensitive in the diagnosis of TB^[16]^ in respiratory specimen. Interferon (IFN)-γ release assays in CSF/blood were applied to aid TBM diagnosis with the sensitive >75%.^[16]^ However, negative results were found in this case on microscopy, culture, GeneXpert, and IFNγ release assays. As GeneXpert test was less sensitive (37% vs 71%) in nonrespiratory specimen 16S rDNA (IS6110-TaqMan assay).^[18]^ 16S rRNA had a 10 to 100 times lower limit of detection for *MBT* than 16S rDNA,^[9]^ which may explain the positive results by 16S rRNA-targeted PCR in CSF.

In conclusion, there should be awareness of the possibility of *P alactolyticus* or *MTB* intracranial infections following tooth extraction.

## Author contributions

**Data curation:** Qin Huang.

**Formal analysis:** Fan Wu.

**Funding acquisition:** Yun Ling, Fan Wu.

**Methodology:** Fahui Dai, Yanling Feng.

**Project administration:** Yun Ling.

**Writing – original draft:** Yixin Liao.

**Writing – review & editing:** Yun Ling, Hongzhou Lu.
